# Human-AI Teaming in Critical Care: A Comparative Analysis of Data Scientists’ and Clinicians’ Perspectives on AI Augmentation and Automation

**DOI:** 10.2196/50130

**Published:** 2024-07-22

**Authors:** Nadine Bienefeld, Emanuela Keller, Gudela Grote

**Affiliations:** 1 Department of Management, Technology, and Economics ETH Zurich Zurich Switzerland; 2 Institute of Intensive Care Medicine Department of Neurosurgery University Hospital and University of Zurich Zurich Switzerland

**Keywords:** AI in health care, human-AI teaming, sociotechnical systems, intensive care, ICU, AI adoption, AI implementation, augmentation, automation, health care policy and regulatory foresight, explainable AI, explainable, human-AI, human-computer, human-machine, ethical implications of AI in health care, ethical, ethic, ethics, artificial intelligence, policy, foresight, policies, recommendation, recommendations, policy maker, policy makers, Delphi, sociotechnical

## Abstract

**Background:**

Artificial intelligence (AI) holds immense potential for enhancing clinical and administrative health care tasks. However, slow adoption and implementation challenges highlight the need to consider how humans can effectively collaborate with AI within broader socio-technical systems in health care.

**Objective:**

In the example of intensive care units (ICUs), we compare data scientists’ and clinicians’ assessments of the optimal utilization of human and AI capabilities by determining suitable levels of human-AI teaming for safely and meaningfully augmenting or automating 6 core tasks. The goal is to provide actionable recommendations for policy makers and health care practitioners regarding AI design and implementation.

**Methods:**

In this multimethod study, we combine a systematic task analysis across 6 ICUs with an international Delphi survey involving 19 health data scientists from the industry and academia and 61 ICU clinicians (25 physicians and 36 nurses) to define and assess optimal levels of human-AI teaming (level 1=no performance benefits; level 2=AI augments human performance; level 3=humans augment AI performance; level 4=AI performs without human input). Stakeholder groups also considered ethical and social implications.

**Results:**

Both stakeholder groups chose level 2 and 3 human-AI teaming for 4 out of 6 core tasks in the ICU. For one task (monitoring), level 4 was the preferred design choice. For the task of patient interactions, both data scientists and clinicians agreed that AI should not be used regardless of technological feasibility due to the importance of the physician-patient and nurse-patient relationship and ethical concerns. Human-AI design choices rely on interpretability, predictability, and control over AI systems. If these conditions are not met and AI performs below human-level reliability, a reduction to level 1 or shifting accountability away from human end users is advised. If AI performs at or beyond human-level reliability and these conditions are not met, shifting to level 4 automation should be considered to ensure safe and efficient human-AI teaming.

**Conclusions:**

By considering the sociotechnical system and determining appropriate levels of human-AI teaming, our study showcases the potential for improving the safety and effectiveness of AI usage in ICUs and broader health care settings. Regulatory measures should prioritize interpretability, predictability, and control if clinicians hold full accountability. Ethical and social implications must be carefully evaluated to ensure effective collaboration between humans and AI, particularly considering the most recent advancements in generative AI.

## Introduction

The rapid development of artificial intelligence (AI) and machine learning offers unprecedented opportunities for supporting physicians and nurses (hereafter clinicians) in a wide range of clinical and administrative tasks [1,2]. Despite these promises, however, integrating AI into clinical practice remains slow, with significant implementation hurdles [3-5].

One key obstacle is failing to account for the broader sociotechnical system (STS) in which humans and AI collaborate. Disregarding the seamless integration of AI with human work practices and the overall systems in which they operate can not only lead to a lack of acceptance [6,7]; but also introduce unwanted errors, patient safety risks, and, in the long run, increase rather than decrease costs [8].

To fully harness the complementarity between humans and machines [9], advancements in machine capabilities must be accompanied by simultaneously building human competencies for successfully collaborating with machines [8]. Contrary to popular belief, more capable systems do not necessarily reduce human effort or errors. Paradoxically, complex systems like AI can translate into higher rather than lower demand for skilled personnel and the need for enhanced expertise, ultimately leading to decreased efficiency and potential performance losses [10]. Furthermore, numerous accidents in aviation, for example, the crash of Air France 447 [11], serve as tragic demonstrations that increased system complexity and a shift from manual to supervisory control are challenging for humans in terms of maintaining situational awareness, the loss of control, or deskilling [12-15].

The lack of transparency in today’s black-box AI systems raises the additional question of who should be responsible for the system’s outcomes. If humans are to maintain overall authority over system goals and their attainment, both accountability and control over system functioning must be afforded to humans, requiring transparency, predictability, and means to influence the systems [16]. In most of today’s AI applications, these conditions are not met, exposing clinicians to significant legal and professional repercussions and raising questions about reassigning accountability to other entities (eg, AI development firms or health insurers) [17].

This study integrates different viewpoints on these issues by data scientists developing AI systems for intensive care unit (ICU) and the clinicians ultimately using these AI systems. We thus tackle the question of how humans can effectively collaborate with AI from a holistic STS perspective [8] and in a context where AI could support overworked clinicians based on AI solutions using high volumes of patient data [18,19].

Contrary to technology-driven initiatives, our human-first approach considers the complementarity between humans and machines to create joint cognitive systems (humans and AI) that achieve better outcomes than humans or AI could achieve on their own [20]. Just because a particular AI application has the potential to augment or automate a given task does not mean that it should do so at all costs. We thus explore the question of social desirability and ethical acceptability of task automation or augmentation, both from the perspectives of clinicians currently performing these tasks and data science experts developing AI solutions.

By defining optimal human-AI teaming in clinical practice and considering the broader sociotechnical context in which AI operates, this study contributes to a more effective, safe, socially acceptable, and ethically sound use of AI in health care. The resulting decision framework can assist hospital managers, policy makers, and legislators in making well-balanced decisions about AI implementation and use to reduce the workload for clinicians and advance the overall quality of care.

## Methods

### Overview

This multimethod study combines (1) a systematic sociotechnical analysis of ICU work tasks with (2) an international Delphi survey among n=19 data science experts to assess optimal levels of augmentation and automation of these tasks; and (3) n=61 semistructured interviews with clinicians exploring their views on AI augmentation and automation.

### Ethical Considerations

Ethics approval was obtained from the local ethics committee at ETH Zurich (number EK 2019-N-51 and EK 2019-N-190), and informed consent was obtained from all participants before data collection.

### Sociotechnical Work Task Analysis

The sociotechnical work task analysis was conducted using COMPASS (Complementary Analysis of Socio-technical Systems), a well-established framework for assessing work tasks and systems based on STS theory [20-22]. Detailed observational notes were analyzed using COMPASS [22] to identify 6 core tasks performed by ICU clinicians (Textbox 1). Observations were carried out as part of a related study [23], during which the first author systematically observed all work-related activities performed by ICU clinicians for 30 morning shifts (8.5 hours each). Additional data were gathered from hospital documents, such as descriptions of job profiles, professional competencies, and organizational charts. Two ICU department heads checked and validated the accuracy and adequacy of the analysis concerning the correctness of medical knowledge and adequate use of terminology [24].

**Core tasks performed by intensive care unit clinicians (based on observation and COMPASS work system analysis).**

- Monitoring patient data (derived from biosensors such as vital signs, parameters from artificial ventilation, or laboratory values).
- Documenting clinical information.
- Analyzing medical data (eg, from reports, articles, test results, or images).
- Prescribing medication or treatment.
- Diagnostic decision-making.
- Interacting with patients.

### Delphi Survey

To assess data science experts’ agreement on the levels of human-AI teaming and technological feasibility to augment or automate each of the 6 core tasks, we conducted an international Delphi survey [25,26]. The Delphi survey is an iterative process method aiming to forecast future (technological) developments and attain consensus among a group of experts regarding questions where there are no clear right or wrong answers and where there is limited or contradictory information. The Delphi method has been shown to outperform other group consensus-finding methods regarding accuracy and efficacy [27], making it a commonly used technique in health information management and health care (eg, [25]).

#### Selection of International Data Science Experts

The quality of Delphi surveys heavily depends on the adequate choice of experts [28]. Therefore, we purposefully selected each expert based on their internationally renowned expertise in the fields of bioinformatics, bioengineering, and health data science. Within these fields, we included academic researchers (professors) from global top-tier universities and data scientists employed by global health care technology manufacturers. Suitable participants were identified through academic publications, participation at conferences, topic-based newsletters [29], and a search of companies and job descriptions on the professional social network LinkedIn, aiming to create a heterogeneous sample regarding geographical regions. Based on the recommended number of participants for Delphi surveys between 10 and 35 [30], we invited 20 data science experts (DS 1-20), out of whom 19 agreed to participate (response rate=95%). Data scientists were 21% female (the relatively low proportion of female experts represents the gender distribution in the field of data science), between ages 29 and 47, and had between 8 and 21 years of experience as professionals in their domain. A total of 37% of experts were employed by global health care technology manufacturers and 67% were employed as faculty of top-tier universities worldwide; 42% of experts were from the AMER (North, Central, and South America) region; 42% from the EMEA (Europe, the Middle-East, and Africa) region; and 16% from the APAC (Asia Pacific, excluding China) region.

#### Survey Development and Data Collection

As an entry point to the Delphi survey and to provide common ground among experts, we used Russel and Norvig’s definition of AI as “machines that mimic cognitive functions that humans associate with the human mind, such as learning and problem-solving” [31] and described each of the 6 core tasks performed by ICU clinicians in short vignettes to illustrate the ICU work system and workflow (Multimedia Appendix 1). Experts were then asked to anonymously select which level of automation and augmentation they deemed best suited to enable safe and effective human-AI teaming for each task from a technological feasibility perspective. The levels of human-AI teaming were developed based on Johnson and colleagues’ taxonomy that goes beyond the traditional levels of automation [32], in that it “more effectively models the technology, the human, and the work [system] together” [33] (Table 1). In addition, data scientists were asked to indicate which of the core tasks performed in the ICU *should or should not* be augmented or automated by AI based on their subject matter expertise, including social and ethical considerations beyond merely considering technological feasibility. For each answer, participants were asked to provide an open-text explanation to justify their choices. The first 3 experts to respond to our initial invitation were chosen to pilot-test the Delphi survey, which resulted in only minor changes. Experts submitted their responses anonymously via email in multiple survey rounds. At each data collection point, 2 participation reminders were sent via email. There were no dropout cases.

Statistical data about the group’s collective choices and the qualitative answers given by data scientists explaining their choices were anonymously fed back to all participants in each consecutive round. As recommended by Sumsion [34], agreements above 70% were considered as consensus. This goal was achieved in round 3 and the Delphi survey was terminated then.

**Table 1:** Results from the Delphi survey—data science experts’ agreement in 3 Delphi survey rounds.

Task	Level 1: AI is unable to significantly augment human performance %^a^			Level 2: AI augments human performance (humans benefit from AI augmentation but are always needed), %				Level 3: humans augment AI performance (AI could perform alone, but human input increases reliability), %				Level 4: AI automates tasks without human input (AI performs reliably; humans serve as troubleshooters), %				Should AI be used?, %	
	1	2	3	1	2	3	1		2	3	1		2	3	Yes		No
Monitoring patient data	—^b^	—	—	—	—	—	—		—	—	63.6		63.6	72.7	92		8
Documenting clinical information	—	—	—	—	—	—	90.1		90.1	90.1	—		—	—	100		—
Analyzing medical data	—	—	—	—	—	—	81.8		90.1	100	—		—	—	92		8
Prescribing medication or treatment	—	—	—	81.8	90.1	90.1	—		—	—	—		—	—	92		8
Diagnostic Decision-Making	—	—	—	81.8	90.1	90.1	—		—	—	—		—	—	53		47
Interacting with patients	90.1	90.1	90.1	—	—	—	—		—	—	—		—	—	—		100

^a^Percentage values are used as a measure of agreement among experts.

^b^Not applicable.

#### Survey Data Analysis

The Qualtrics survey web-based platform [35] was used to develop the survey and collect data. SPSS (version 24; IBM) was used for statistical data analysis.

### Interviews With ICU Clinicians

To examine ICU clinicians’ perspectives on the social and ethical implications of AI-enabled automation and augmentation technologies, we conducted 61 semi-structured interviews as part of a larger research project [36]. Interview questions related to clinicians’ vision of future human-AI teaming solutions and the distribution of control and accountability (Multimedia Appendix 2). Interviews were conducted in private offices, lasted between 60 and 90 minutes, and were audio-recorded and manually transcribed ad verbatim.

#### Selection of Clinicians

In line with grounded theory [37], we used a theoretical sampling approach to include both professional groups of ICU physicians and nurses (25 physicians and 36 nurses). Clinicians were 56% female and had between 2 and 30 years of experience after completing their initial education as a registered nurse (RN) or board-certified ICU physician. All informants were directly involved in care delivery. We gave each informant a code showing their professional position (attending physician [AP], resident physician [RP], and RN) and personal identifier (1–61).

#### Interview Data Analysis

The analysis of the interview content was conducted following the grounded theory methodology [37]. The principal investigator engaged in the process of open coding, methodically labeling each discrete conceptual unit within the interview transcripts. This procedure entailed the aggregation of related codes into broader categories and themes. Subsequently, a review of the related textual segments was performed to verify the consistent alignment of the data with the identified themes, a step that is pivotal for maintaining the integrity of qualitative inquiry [38]. The software MAXQDA (version 2024; VERBI GmbH) was used to facilitate qualitative data analysis [39]. The presentation of the qualitative findings adheres to the protocols endorsed by the Academy of Medicine [40].

## Results

### Data Science Expert Perspective

Table 1 summarizes the results from the Delphi survey. Data science experts’ assessments about the technological potential to augment or automate each ICU task were quite similar from the start, and increasing consensus between 72.7% and 100% was reached across the 3 Delphi survey rounds. Data science experts also showed high levels of agreement (92%-100%) about which of the 6 tasks *should or should not* be augmented or automated by AI based on social and ethical considerations, except for one task: For the task “diagnostic decision-making” 53% of data science experts recommended the use of AI, whereas 47% did not.

In what follows, we provide a detailed account of how the consensus-building process among data science experts unfolded as part of the Delphi survey process. For each task and level of human-AI teaming, we provide illustrative statements by data science experts justifying their choices.

#### Monitoring Patient Data

In the first round, 63.6% of data science experts agreed that AI could eventually fully automate this task and that human assistance would bring no further benefits (level 4). This result remained the same also in the second round but increased to a 72.7% consensus in the third round. One data science expert justified this choice by stating:

Large longitudinal data like heart rate, BP, etc., are much harder to interpret for humans. It's like weather forecasting where you have so many data points and supercomputers can more accurately model weather than humans. Even the most experienced clinicians are overwhelmed with today’s data overload and since human monitoring is not feasible at all times, full automation without human interference is really the best option (DS 17).

A total of 27.3% of experts selected the design choice “AI could do it, but humans increase reliability” in the first and second rounds:

Monitoring of the signs is possible to learn by ML; however, having human help when an obvious mistake happens increases efficiency and reliability and seems unavoidable. AI could not do it as reliably and reproducibly as doing it together with the human (DS 05).

Regarding the question as to whether “monitoring patient data” *should* be automated by AI (level 4), all but one expert (92%) agreed that there were no social or ethical concerns:

Automated monitoring is bound to be better than what there is now. Even if the system fails at times, it’s not that patients will die from this (DS 11).

#### Documenting Clinical Information

In the first round, 90.1% of experts agreed that AI could eventually automate this task, but human assistance would increase reliability (level 3). Expert agreements remained stable throughout all 3 rounds. The following argument was given by a data science expert, further explicating their choice:

There are a lot of approaches that involve humans in the loop such as active learning/weak supervision that can greatly augment the reliability of AI techniques used for these types of tasks. With greater data standardization and curated datasets, the goal of greater automation becomes more and more feasible but for now, physicians will have to be in the loop (DS 07).

Also, 100% of experts agreed that this task *should* be augmented by AI, for instance, as one data science expert argued:

That’s [the task of documenting clinical information] what [clinicians] don’t like doing and where AI can really help so they can focus on more interesting things (DS 15).

#### Analyzing Medical Data

Already in round one, most data science experts (81.8%) agreed that AI could eventually do this task autonomously but that human assistance would increase reliability (level 3), for instance, stating that:

So many fields are already doing automated reporting and with humans in the loop, this becomes easier and easier for AI systems to automate and for medical professionals to interpret. Again, not fully autonomous but moving closer on the continuum towards it (DS 09).

Two data science experts adopted the consensus opinion in the second and third rounds of the Delphi process, resulting in a 100% agreement. One of them provided the following explanation for changing from level 4 to level 3:

I agree. The process of automatically producing “draft analysis reports” can be fully automated but if we take human sense-making as a kind of “assistance” then I would also say that this [level 3] is the right choice (DS 18).

Furthermore, 92% of experts agreed that there were no social or ethical concerns and that this task *should* be augmented by AI (level 3).

#### Prescribing Medication or Treatment

In the first round, 81.8% of data science experts agreed that this task could be augmented by AI but would always require humans in the lead (level 2). This opinion increased to 90.1% in rounds 2 and 3. The following arguments were given by data science experts, explaining their choices:

Although automated diagnostics and algorithmic medication prescriptions have become extremely accurate because mistakes are very costly, a medical professional should always review and approve the suggestions produced by the software (DS 10).

One expert (9.1%) argued that AI could perform this task with human assistance (level 3) and did not change his or her opinion throughout the Delphi survey process based on the following explanation:

I don’t think human oversight is always required. It will be like how self-driving cars are gradually phasing into our society as well. There are certain use cases where automated diagnostics can yield greater results in a well-designed system that triages events earlier in the healthcare system and prevents issues later downstream that have bigger healthcare impacts in terms of patient health and costs (DS 19).

In total, 92% of data science experts agreed that this task *should* be augmented by AI as long as humans are accountable and in the lead (level 2):

AI tools will never be ready to prescribe meds without a human in the loop simply because they don't take legal liability for the decisions which ultimately will have to be made by the physician (DS 06).

#### Diagnostic Decision-Making

In the first round, 81.8% of data science experts indicated that AI could augment this task, but humans would always be required (level 2), and in the second and third rounds, 90.1% selected this choice. As one data science expert stated:

I think our [AI] solutions are far too narrow for this type of decision-making. They [AI solutions] can assist but need human input always (DS 05).

Two experts (18.2%) chose level 3 for this task in the first round stating that:

Diagnostics and prognosis can be automated really well with AI/ML but depends on how it is accepted. But in my opinion, having 100% human supervision defeats the purpose of AI/ML (DS 16).

Concerning social and ethical considerations 47% of data science experts believed that diagnostic decision-making *should not* be augmented by AI, even if it was technologically feasible to do so:

I strongly believe that for as long as we [data scientists] are still struggling with issues of bias, transparency, and equity etc., we should shy away from such consequential tasks [diagnostic decision-making]. ML can be utilized in the form of a “recommendation engine”, but doctors should never rely on it fully (DS 19).

Besides the ethical issues of bias, transparency, and equity, some data science experts also felt that using AI in diagnostic decision-making would undermine physicians’ role identities, which could be problematic.

This is what being a doctor is all about. Applying one’s knowledge and experience to diagnose patients is the very core of medicine. It would be like messing with the Hippocratic oath and taking away the reason why they chose to become a doctor in the first place (DS 08).

#### Interacting With Patients

With a strong 90.1% agreement in all 3 rounds and no changes throughout, experts believed that the task of patient interaction cannot be augmented or automated by AI.

AI/ML technologies simply cannot properly mimic human soft skills which are essential in these sorts of interactions (DS 13).

Only one expert argued that AI could augment humans in their patient interactions (level 2) by stating:

In most cases, it is challenging for AI/ML to interact empathically with patients, but there are some new use cases with promising results that could potentially augment this task (DS 07).

However, regardless of the technological possibilities of AI mimicking empathic patient interactions, all data science experts (100%) agreed that AI *should not* be used to interact with patients, mainly based on social concerns and what it means to be human:

Medicine is all about empathy, one human caring for another [...]. Therefore, [interactions with patients] should never be replaced by an AI [ro]bot or the like (DS 11).

The Role of AI is to provide more time for physicians to do what this empathy-driven field requires most: human interaction and connection. No AI should interfere with that (DS 17).

### Clinician Perspective

In the following, we report clinicians’ views on how to best team up with AI when augmenting or automating ICU tasks and conclude with a brief comparison between the 2 stakeholder groups.

#### Monitoring Patient Data

Like data scientists, clinicians acknowledged the benefits of automating the monitoring task. Many clinicians, however, pointed out the need for highly reliable systems so they would no longer have to constantly supervise AI, which defeats the purpose of gaining time and increasing efficiency:

Taking over the monitoring [task] would really save us time and reduce our workload. But of course only if the AI is so good that we don't have to constantly monitor it. Because until now, with AI [systems], we still have to do the monitoring so it is more like a double effort and I actually have less time for the patient (RN 43).

#### Documenting Clinical Information

Clinicians perceived the documentation task as “the most time-consuming [task] of all,” imagining a future where AI could “take away that burden” (RP 12) and welcoming high levels of automation for this task. As one AP explained, their future roles would ideally consist of merely checking for potential errors in AI-produced documents:

This [automation of clinical documentation] would be a huge relief, especially for residents because they are the ones who spend the most time on administrative tasks. I see them sit here [in the ICU] for hours on end way past they clock out [after the end of their shift] and I think to myself, “All those years of medical school to do what - office work!” (AP 39).

#### Analyzing Medical Data

Clinicians saw immense potential in using AI to aid with the analysis of medical data, but only if they could “stay in the driver’s seat.” Most clinicians said they would always want “to decide whether to follow the advice [given by AI] or not” (AP 11). One nurse gave the example of using AI to analyze medical data so they could foresee the risk of delirium in ICU patients and initiate prophylactic actions before problems occurred:

All the vast amounts of data that we can use to predict the onset of conditions such as delirium. That would be brilliant because delirious patients have a considerably higher mortality rate and are very resource-intensive. In some ICUs, [the use of AI to analyze medical data] is already available, for instance, to predict sepsis and so on. A lot is going on at the moment and I see a huge opportunity [for AI] to recognize all these risks in patients so that we can simply say, “Oh yes, that makes sense” and take prophylactic action (RN 36).

#### Prescribing Medication or Treatment

Similar to data scientists, clinicians realized AI’s potential to augment the task of prescribing medication and treatment, but they stressed the importance of keeping the ultimate decision power in their own hands. As one RP explained this important precondition of using AI for this task:

Yes, of course, an AI system that can provide us with information on adverse drug interactions or patient-specific intolerance, for example, would be great because we humans are much more prone to error for these kinds of tasks than machines. This also goes in the direction of personalized medicine and that is the future for sure. But one thing that must not happen is that the system then directly initiates a therapy, that would be too dangerous (RP 58).

One use case that was discussed intensively by clinicians was the use of AI to assist decision-making around which treatment patients should receive based on ethical considerations and long-term prognosis:

AI could help us a lot in deciding whether and if so which therapy really makes sense for a patient in terms of prognosis and quality of life long-term. Now, we have interdisciplinary ethics boards for this [decision], but the outcome for each patient is mostly unclear. If AI could show all the facts and the prognosis and perhaps also visualize them, our decisions would ultimately be more ethical, objective, comprehensible, and better in terms of health economics for sure (RN 30).

#### Diagnostic Decision-Making

Unlike data scientists believing that clinicians would be worried about their loss of status or power, not a single clinician worried about themselves. Rather than focusing on the changes AI would incur in their professional lives, clinicians focused solely on the benefits AI would bring to their patients. As one AP explained, putting patient benefits front and center is what matters most in any decision they make:

What’s important here is not whether we like [the changes] or not but how the patient can benefit from [AI solutions]. The goal should be that AI can help as many patients as possible. I mean it’s well known that it is more difficult for us [humans] to diagnose rare diseases and that we have all kinds of cognitive biases. That’s where I see the big potential of AI, that it can help [improve diagnostic accuracy] (AP 01).

Unlike data science experts, issues relating to biased or unfair AI algorithms did not feature in clinicians’ answers. Instead, clinicians highlighted how important it was to “see every patient as a unique human being” and to assess each patient “holistically including individual, social, and environmental factors” (RN 45):

Diagnostic support from AI is great. But where I would have problems is if every patient was treated in the same standardized way and we could no longer have a say. Because every patient is unique; one patient feels fine with a blood pressure of 60 and another patient is lying flat on the floor already. Medicine cannot be generalized, it cannot be standardized following some statistical norms, that would be a setback (RN 43).

#### Interacting With Patients

Clinicians agreed with data scientists on the importance of social interaction, empathy, and human connection in patient interactions. They too were unable to imagine a future where AI would interact socially and demonstrate human-level empathy with patients:

It has been proven that people need human connection and closeness to get well. Our social skills, our empathy, the care received from human to human are enormously important for overcoming even the most difficult crisis situations. I can't imagine that an AI system, a robot, will ever be able to do this. Especially not here in the ICU where patients are always on the border between life and death (RN 36).

Furthermore, interactions with patients were seen as one of the core tasks why clinicians had chosen their profession and one that they hoped they could invest more time in, thanks to AI augmentation and automation:

The human aspect in nursing is actually the reason why I chose this profession. I think it would be incredibly hard for me if it was just a case of operating the AI, maybe turning the patient to his side once a shift and that would be it. So that's what I'm hoping for with AI, that we'll find time again to care for our patients more closely, to talk to them or their relatives in peace, to do all the actual caring again. Unfortunately, there's really not enough time for that anymore these days (RN 17).

To summarize, clinicians’ and data scientists’ perspectives regarding the optimal levels of human-AI teaming, and ethical and social concerns were very similar across all tasks. However, clinicians provided a more nuanced picture regarding the effects different levels of augmentation and automation would have on their ability to validate AI’s performance. Specifically, they argued for higher rather than lower levels of automation for the tasks of monitoring and documentation, provided that AI performance was highly reliable. Clinicians agreed with data scientists that they would need to retain the ultimate control and decision-making power for the tasks of analyzing medical data, prescribing medication or treatment, and clinical decision-making, but not because they feared losing professional status or purpose but because they were worried about patient safety. Clinicians worried less about bias or inequity in AI but more about standardization and failing to see each patient as a unique human being, that is, that the recommendation is plausible given a specific patient situation in the clinical context. Finally, for the task of interacting with patients, both stakeholder groups were clear about not wanting AI to interfere because they both valued “real” human empathy and human-to-human connection.

## Discussion

### Principal Findings

In this study, we identified 6 core tasks characterizing the work in hospital ICUs and assessed optimal levels of human-AI teaming for each task, considering data science experts’ and ICU clinicians’ perspectives. Such a human-centered assessment based on STS theory contributes to the ongoing debates about AI use, as well as the social and ethical implications of AI in health care in the following important ways.

First, contrary to the predominant focus on what AI can and will be able to achieve, our approach considers the strengths and weaknesses of both humans and AI in a complementary fashion. Such an approach reduces the risks of misalignment and aims at achieving better overall system outcomes than humans or AI could achieve alone [8]. The ICU context served as an ideal context due to the availability of large amounts of patient data and the need for technological support. The methodology itself can be used in any work system in health care and beyond.

Second, whereas the current consensus on the future of AI in health care suggests that augmentation is always better than automation, the results from our Delphi study and the responses from clinicians themselves paint a more nuanced picture. Augmentation (levels 2 and 3) was considered the ideal form of human-AI teaming for 4 out of 6 tasks, but only if certain requirements were met. From data scientists’ perspectives, these requirements include transparency, predictability, and sufficient means to influence the system [16,21]. Currently, the goals of higher transparency and predictability are not always attained due to black-boxed AI systems. Clinicians said they would not need to understand the AI algorithms as long as they provided interpretable results (eg, people can drive a car without knowing how the engine works). Involving clinicians in the co-design of interpretable rather than fully transparent systems could thus be a solution to solving the explainable AI conundrum [41]. Given the high safety risks for patients, these considerations are particularly important for “diagnostic decision-making,” “prescribing medication or treatment,” and “analyzing medical data.” Also, both stakeholder groups agreed that at levels 2 and 3, both the control over and responsibility for system outcomes must reside with clinicians.

If full human control is not possible (due to a lack of interpretability or because the necessary skills or knowledge are absent or lost over time), legal responsibility would need to shift to another entity (eg, AI providers). In cases where AI applications advance to a level of robustness and near-perfect reliability yet human control is not guaranteed, higher levels of automation can increase overall system safety and efficiency [14]. For “monitoring patient data,” data science experts and clinicians chose level 4 automation because it is not humanly possible to continuously monitor automated systems.

Looking into the future and considering the fast-moving developments in generative AI (GenAI) applied to health care [42], tasks such as “documenting clinical information” or “analyzing medical data” could benefit from higher levels of automation but also introduce new ethical and security risks [43]. Because these models are capable of combining multiple modalities (text, image, code, or video), AI applications may soon no longer be restricted to performing single tasks. Contemplating the state-of-the-art performance of such future AI applications on a wide variety of problems [44,45], they have the potential to automate tasks such as “documenting clinical information” by integrating and conjointly analyzing laboratory, visual, and patient history data. Also, while such systems currently do make mistakes, they are also able to self-correct when prompted to do so [2].

Third, for the task of interacting with patients, both data science experts and the clinicians in this study believed AI should not assist humans even if it were technologically feasible. They unanimously agreed that compassion and empathy lie at the core of the physician-patient or nurse-patient relationship and that these innately human qualities should remain in the sole hands of humans. Intriguingly, ChatGPT (version 4) from OpenAI [46] was recently rated as more “empathetic” than human physicians by a panel of expert physicians assessing physician-patient interactions posted on social media [47]. GenAI could be used to assist clinicians in brainstorming ideas on how to best prepare for difficult patient conversations or to simulate which questions patients are likely to have. This is not to say that clinicians lack these skills altogether, but that they often lack the time to prepare for challenging patient interactions [2]. After all, if AI can help free up time for tasks spent away from patients, clinicians’ number one desire—(re)gaining time spent with their patients—could finally be realized [48].

Finally, the question of whether “diagnostic decision-making” should be augmented and automated by AI or not revealed the largest disagreement both within the groups of data science experts (53% yes vs 47% no) and across data science experts and clinicians. Contrary to the 47% of data science experts who believed AI should *not* be used for this task, all clinicians in our study thought that it should, as long as they could retain the final decision-making power. Concerns about the bias or fairness of AI algorithms did not feature in clinicians’ responses. Instead, clinicians stressed the importance of making holistic patient assessments and that every patient is a unique human being with specific circumstances that must be considered. Moreover, some data science experts were concerned that AI augmentation or automation of the diagnostic decision-making task would threaten clinicians’ role identity and professional sense of meaning and that AI should not be used for these reasons. Clinicians, however, put the benefits AI could bring to their patients above any such concerns. Finally, from a sociotechnical perspective, one important question remains: if AI applications would one day be capable of augmenting diagnostic accuracy beyond human levels, how will clinicians retain the ability to build the necessary medical expertise to control the results? Also, with increasingly accurate and capable systems, the risk of overreliance is likely to increase, which could lead to long-term consequences of deskilling and loss of expertise [49].

### Implications for Policy and Practice

**Figure 1:**
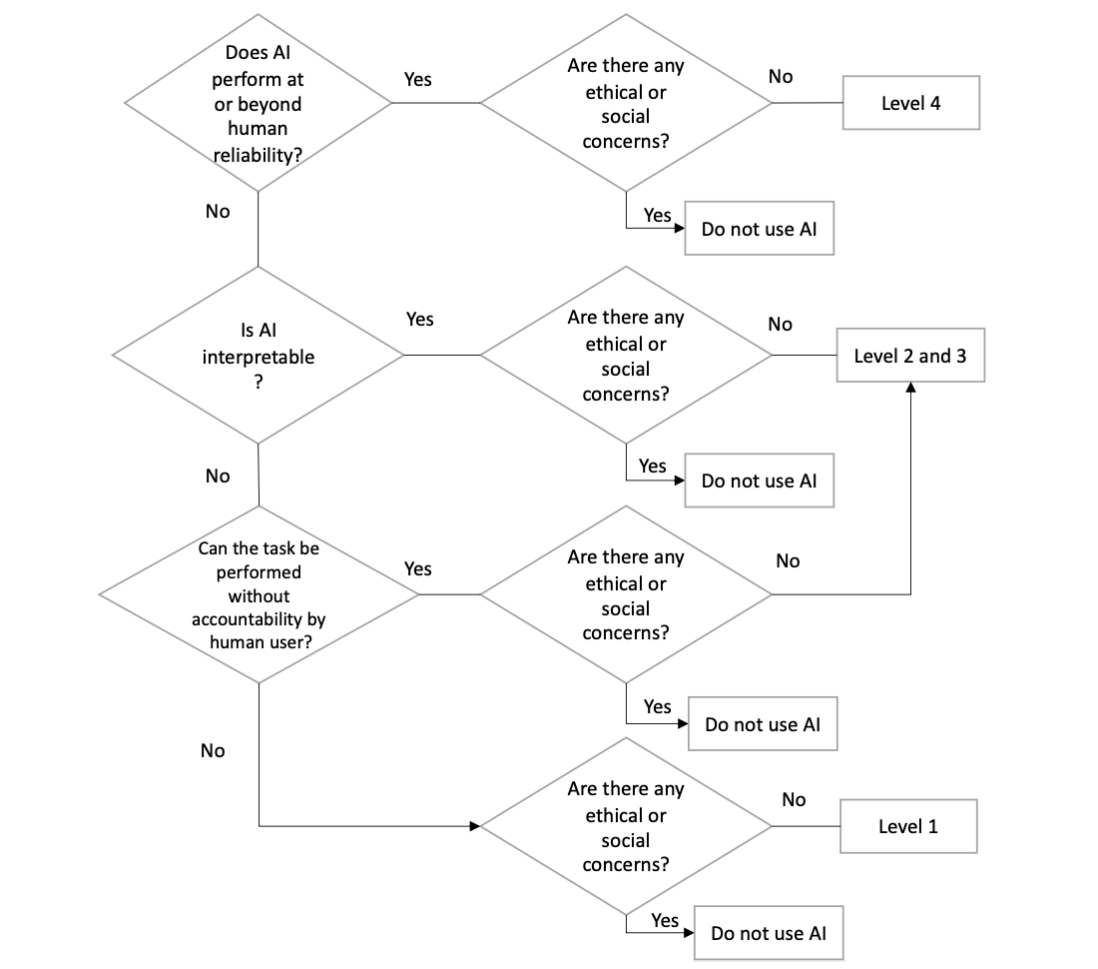
Decision flowchart summarizing recommended levels for human-AI teaming based on study results and STS literature. AI: artificial intelligence; STS: sociotechnical system.

Based on these results, Figure 1 shows a decision diagram aimed at assisting hospital managers, IT managers, and policy makers in assessing optimal levels of human-AI teaming that enable safe and effective AI use. The decision process is based on the assumption that reliability, interpretability, and accountability are key factors in defining optimal levels of human-AI teaming by augmenting and automating a task. Furthermore, regardless of technological capabilities, the question of whether there are any ethical or social concerns to be considered should always be asked. As depicted in Figure 1, if AI reliability is near perfect and there are no ethical and social concerns, level 4 automation is advised. If AI does not (yet) perform highly reliably but system interpretability is granted—or if accountability is shifted away from human users—level 2 or 3 is recommended. Finally, if AI systems are neither reliable nor interpretable and accountability remains with human users, level 1 is advised, or AI should not be used. Textbox 2 summarizes this study's key takeaways and implications for policy making and practice.

**Implications for policy and practice.**

1. Adopting an STS perspective can prevent costly misalignment between AI, humans, and their work and facilitate implementation into clinical practice.
2. Optimal human-AI teaming is based on considering the strengths and weaknesses of humans and AI in a complementary fashion, thus creating better outcomes than either one could achieve alone.
3. Clinicians should be involved in co-designing future AI applications (eg, [41]) to leverage the complementarity of humans and AI and to continuously monitor human-AI teaming effectiveness post-implementation.
4. Clinicians need specific knowledge, skills, and attitudes to effectively work in human-AI teams; human-centered interaction design is not enough.
5. At the level of augmentation (levels 2 and 3), clinicians must be enabled to understand which data are used to make predictions, interpret results within the clinical context, and have adequate means of controlling the system.
6. If AI achieves near perfect (human-level or above) reliability yet the conditions 1-5 are not met, level 4 automation can create higher safety and efficiency.
7. All relevant stakeholders (eg, clinicians, patients, regulators, management, and patient safety experts) must decide whether AI should or should not be used based on social and ethical considerations beyond technological feasibility.
8. Regulatory foresight must address the question of control and accountability and reconsider liability assignments and insurance arrangements.
9. The rapid evolution of AI solutions needs frequent reevaluations of the STS.
10. Policy around AI-enabled in-context learning is needed, especially regarding new generations of generative AI and LLMs [1].

### Limitations and Future Research

Several limitations must be considered when interpreting the results of this study. First, as we used the example of ICUs to forecast the future use of AI to augment or automate various tasks, the peculiarities of other medical domains in terms of tasks, level of risk, and ethical concerns should be specified in future research.

Second, although the Delphi technique has proven to be an ideal method for developing probable future scenarios based on experts' present knowledge and outperforms comparable interactive group forecasting techniques [27], the actual realization of the forecast depends on multiple other factors, such as the availability of resources, legal frameworks, or the acceptance by clinicians. Although the clinicians in our study agreed with data science experts about the optimal levels of human-AI teaming for most tasks, there were some differences, such as the importance of considering a patient situation holistically (eg, social context or environmental factors), beyond measurable data. Also, in the rapidly evolving landscape of AI, expert assessments may change and will need to be adapted over time. Future research, and ideally also policy making, should adopt our proactive approach and regularly ask a panel of data science experts, clinicians, and additional stakeholders, such as health insurers or hospital management, about their views. In that way, all stakeholder groups can be one step ahead of the upcoming technological change. This presents an alternative route to the currently predominant technology-driven approaches to AI implementation in health care.

Third, cultural aspects are known to influence people's perceptions regarding the social and ethical considerations of AI use [50]. For this reason, we paid special attention to including a broad sample of data science experts from the AMER, EMEA, and APAC regions. In this study, we found no notable differences between these geographical areas, but we acknowledge that we failed to recruit data science experts from China and Africa. Future research should involve China and African countries, especially. Also, the clinicians in our study, although representing multiple nationalities, are not a fully representative sample of the global ICU clinician population. Even though most ICUs adhere to similar standards and work tasks, there may be other factors, such as the availability of resources, to consider.

Lastly, despite the widespread use of the Delphi technique, particularly in medical informatics research [25,26], several concerns have been raised. Anonymity, a central characteristic of the Delphi technique, is intended to promote unbiased assessments from experts by eliminating social desirability bias. Nevertheless, it has been argued that anonymity may lead to hasty judgments, as experts feel relieved from the responsibility of defending their responses [51]. Additionally, the Delphi technique necessitates the disclosure of interim results in each round to facilitate the generation of a group consensus, which some scholars contend compromises independent judgment because this disclosure may exert social pressure on outliers to revise their assessments, thereby promoting group conformity instead of genuine changes in opinion [52]. Hence, as noted by McKenna [53], group consensus should not be regarded as the only “correct answer,” but seen as a way to structure the discussion among experts in domains where no right or wrong answers exist. In this study, we believe we achieved this goal by additionally asking experts to provide open-text answers explaining their motivation to change previous ratings.

### Conclusions

With the overall aim of enabling safe, socially accepted, and ethically sound use of AI to reduce workload and improve quality of care, this study offers valuable insights into the potential of human-AI teaming in ICUs and wider health care settings. By adopting an STS perspective, we emphasize the importance of considering human and AI strengths and weaknesses in a complementary fashion to optimize outcomes and minimize misalignment. The findings challenge the prevailing notion that augmentation is always preferable to automation by demonstrating a more nuanced picture of the ideal interaction levels for each task.

We propose several key principles for implementing AI in health care for policy and practice, for example, incorporating user-centered co-design, promoting transparency and predictability, and ensuring control over AI systems in cases where accountability resides with users. Moreover, assessing ethical and social implications when deciding on AI applications is important, especially considering the promising new developments in GenAI. Regulatory foresight and knowledge about optimal fit between work tasks and levels of human-AI teaming will be crucial in shaping the future of AI in health care and beyond.

## Appendix

Multimedia Appendix 1 Delphi Survey Vignettes to describe ICU tasks and workflow. ICU: intensive care unit.

Multimedia Appendix 2 Interview guidelines.

## Abbreviations

AI: artificial intelligence
AMER: North, Central, and South America
AP: attending physician
APAC: Asia Pacific, excluding China
EMEA: Europe, the Middle-East, and Africa
COMPASS: Complementary Analysis of Socio-technical Systems
GenAI: generative AI
ICU: intensive care unit
RN: registered nurse
RP: resident physician
STS: sociotechnical system
