# Supplementary material for: Human-AI Teaming in Critical Care: A Comparative Analysis of Data Scientists’ and Clinicians’ Perspectives on AI Augmentation and Automation
Source: J Med Internet Res. 2024 Jul 22;26:e50130. doi: 10.2196/50130 (PMC11301121; doi:10.2196/50130)
Supplement: Multimedia Appendix 1 [file jmir_v26i1e50130_app1.pdf]

Appendix A

Delphi Survey Vignettes to describe ICU tasks and workflow

A) Overall work system and workflow in the ICU

The intensive care unit (ICU) represents a complex and dynamic work environment dedicated to the management of critically ill patients, necessitating a multidisciplinary approach and the employment of sophisticated technologies used for monitoring, treatment, and basic life support. This setting demands extensive, well-coordinated communication across a skilled interdisciplinary team, including ICU physicians and nurses at various levels of expertise, who operate within a structured hierarchy ranging from attending physicians to resident physicians and registered nurses. ICU teams typically work in 3 shifts of around 8-9 hours each. Typically, patients in the ICU are bed-bound for an average duration of two to three weeks, often unconscious or under heavy sedation, with the medical team vigilantly overseeing their vital signs and therapeutic progression. ICU physicians and nurses must respond instantly to the frequent variances and disturbances that characterize the demanding nature of ICU care. Amongst a wide range of ICU tasks performed, we have identified six core tasks, which we describe in detail next.

B) Six core tasks in the ICU

1. Monitoring patient data:

This task includes monitoring vital signs such as body temperature, blood pressure, pulse, respiration rate, quantification of all fluid intake and output, and often intracranial pressure and/or daily weight to detect changes in the patient's health condition status and to predict upcoming problems.
Cognitive abilities needed to perform this task include:
Processing information (= compiling, coding, categorizing, calculating, or verifying data)
Information ordering (= arranging data into patterns according to specific rules)
Sense-making (= combining and organizing information into meaningful patterns)
Flexibility & speed of closure (= detecting a known pattern that is hidden in other distracting material)
Perceptual Speed (= quickly and accurately comparing similarities/differences among sets of data)

2. Documenting medical information:

This task enables the continuity and coordination of care across interdisciplinary ad hoc teams working in the ICU and should facilitate communication between different healthcare professionals. Documenting medical information serves as a basis for clinical decision-making and risk assessment and can be done either manually (in files and charts) or digitally (electronic health record systems EHR) a patient's demographic information, medical history, examination findings, diagnosis, detailed treatment history, and future treatment plan, clinical interventions and outcomes, medication administered, prescribed or renewed and any drug allergies, resuscitation status, risk scores, documentation of communications with patient and family/friends. It also serves as a record of patient care for audits and in case of incident investigations.
Cognitive abilities needed to perform this task include:
Processing information (= compiling, coding, categorizing, calculating, or verifying data)
Information ordering (= arranging data into patterns according to specific rules)
Sense-making (= combining and organizing information into meaningful patterns)
Written comprehension & expression

3. Analyzing medical data:

This task relates to ICU clinicians analysis of any type of medical data used to inform diagnostic or treatment decision-making such as data from imaging (radiology, pathology, video recording), cellular or molecular laboratory data, physiologic data from devices, data from medical reports, and test results.
Cognitive abilities needed to perform this task include:
Processing information (= compiling, coding, categorizing, calculating, or verifying data)
Information ordering (= arranging data into patterns according to specific rules)
Sense-making (= combining and organizing information into meaningful patterns)
Deductive Reasoning (= applying general rules to specific problems to produce answers)
Inductive Reasoning (= combining pieces of information to form general rules also among seemingly unrelated events)
Written comprehension & expression

4. Prescribing medication or treatment:
This task includes defining which medication or treatment is suitable, calculating the patient-specific dosage, administration protocol, and duration of a medication or treatment, and checking for drug allergies or negative interaction effects between multiple drugs or treatments.

Cognitive abilities needed to perform this task include:
Processing information (= compiling, coding, categorizing, calculating, or verifying data)
Information ordering (= arranging data into patterns according to specific rules)
Sense-making (= combining and organizing information into meaningful patterns)
Written comprehension & expression

5. Diagnostic Decision Making:
Considering the relative costs and benefits of potential actions by using logic and reasoning and choosing the most appropriate diagnoses. A wide array of medical information (see above), laboratory tests, imaging tests (e.g., x-ray, CT scan), or endoscopy may be used to assist diagnostic decision-making.
Cognitive abilities needed to perform this task include:
Processing information (= compiling, coding, categorizing, calculating, or verifying data)
Deductive Reasoning (= applying general rules to specific problems to produce answers)
Inductive Reasoning (= combining pieces of information to form general rules also among seemingly unrelated events)
Memorization (= remembering information such as words, numbers, pictures, and procedures)
Fluency of Ideas (= ability to come up with a number of ideas about a topic)
Originality (= ability to come up with unusual or clever ideas about a given topic or to develop creative ways to solve a problem)

6. Patient interactions:
This task relates to the 1:1 interactions with patients including those who are deprived of speech because they are sedated and/or mechanically ventilated or unconscious.
Cognitive abilities needed to perform this task include:
Processing information (= compiling, coding, categorizing, calculating, or verifying data)
Oral expression & comprehensionSocial Perceptiveness (= being aware of others' reactions and understanding why they react as they do)
Demonstrating empathy for others
