# Supplementary material for: Human-AI Teaming in Critical Care: A Comparative Analysis of Data Scientists’ and Clinicians’ Perspectives on AI Augmentation and Automation
Source: J Med Internet Res. 2024 Jul 22;26:e50130. doi: 10.2196/50130 (PMC11301121; doi:10.2196/50130)
Supplement: Multimedia Appendix 2 [file jmir_v26i1e50130_app2.pdf]

Appendix B

Interview Guidelines

Icebreaker: Example Case Study

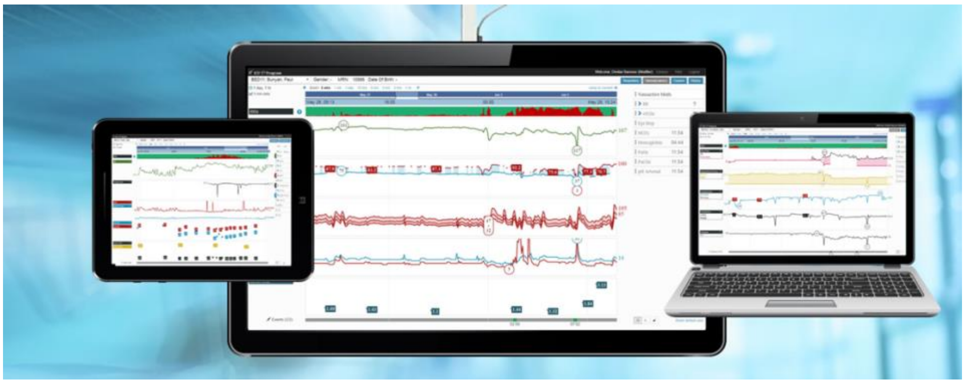

[Extract from http://www.viscomp.etiometry.com/our-platform/t3-visualization/]
The T3 Data Aggregation & Visualization tool by © Etiometry is an FDA-cleared software using machine learning-based DNN (Deep Neural Networks). The application collects, visualizes, and stores ICU data in near real-time. It enables the automatic collection, consolidation, normalization, time synchronization, and binding of data from multiple sources to support patient care. T3's graphical user interface seamlessly delivers data collected from patient monitors, ventilators, and laboratory systems as well as new information produced by Etiometry algorithms to anywhere within various clinical workflows via any popular web browser. Its intuitive visualization synthesizes patient data in a manner that reduces the impact that the limitations of human factors place on the efficient interpretations of multiple streams of patient data within the ICU decision-making process.

1. **Question Category I: Opening**

1.1. What effect does this case study example have on you?

2. **Question Category II: Future vision of human-AI teaming**

2.1. Thinking about your everyday work as a doctor or nurse in an ICU, how do you imagine working with these or similar AI applications in your day-to-day work?

3. **Question Category III: Control and Accountability**

3.1. In these new roles of collaborating with AI, who would have control of what?

3.1.1. Why?

3.2. Who would be responsible for what?

3.2.1. Why, why not?
